# Broadly neutralizing monoclonal antibodies derived from mRNA LNP immunization exhibit potent neutralizing ability against JN.1, KP.3.1.1 and XEC new Omicron variants

**DOI:** 10.1099/jgv.0.002251

**Published:** 2026-04-24

**Authors:** Hsiao-Ling Chiang, Hsiu-Ting Lin, Wan-Yu Chen, Kang-Hao Liang, Ruei-Min Lu, Han-Chung Wu

**Affiliations:** 1 Biomedical Translation Research Center (BioTReC), Academia Sinica, Taipei, 11529, Taiwan, ROC; 2 Institute of Cellular and Organismic Biology, Academia Sinica, Taipei, 11529, Taiwan, ROC

**Keywords:** BA.5, XBB.1.5, neutralizing antibody, severe acute respiratory syndrome coronavirus 2 (SARS-CoV-2), single B-cell antibody technology, XEC

## Abstract

Since the emergence of severe acute respiratory syndrome coronavirus 2 Omicron, dramatic changes in the receptor-binding domain have allowed for virus escape from many therapeutic antibodies. Development of antibodies effective against current viral strains is, therefore, necessary to provide useful clinical treatments and can also yield fundamental information about viral neutralization. Here, we utilized single B-cell antibody technology to isolate and characterize neutralizing antibodies from splenocytes of mice immunized with mRNA-LNPs. With this approach, we identified five BA.5 and four XBB.1.5 neutralizing chimeric antibodies (ChAbs). The identified ChAbs, BA.5-ChAb-41, XBB.1.5-ChAb-17 and XBB.1.5-ChAb-26, each potently neutralized BA.5, XBB.1.5 and JN.1 pseudotype viruses with low IC_50_ (1.9, 2.3 and 10.93 ng ml^−1^). Furthermore, XBB.1.5-ChAb-26 showed neutralizing capability against the current JN.1-related variants, KP.3.1.1 and XEC. By performing single amino acid substitution of the receptor-binding motif (RBM), we observed that the epitopes of BA.5-ChAb-41 and XBB.1.5-ChAb-17 include Y453 and R498 in the RBM. Moreover, XBB.1.5-ChAb-26 epitope includes Y453 and T500, which may contribute to its ability to neutralize JN.1, KP.3.1.1 and XEC variants. In summary, our approach allowed us to efficiently screen for highly functional antibodies, and we further identified critical residues in the epitopes to aid in the design of therapeutic virus-neutralizing antibodies.

## Data Summary

One supplementary figure and two supplementary tables are avalaible with this article. GenBank accession numbers of Surface glycoprotein (Spike protein) of SARS-CoV-2 variants used in this study are available in Table S1.

## Introduction

The severe acute respiratory syndrome coronavirus 2 (SARS-CoV-2) pandemic has resulted in millions of infections and deaths, and it has severely affected healthcare systems around the world. Due to the continuous evolution of SARS-CoV-2, new variants have become more transmissible and often exhibit immune evasion. The previous variants of concern (VOCs) – Alpha (B.1.1.7), Beta (B.1.351), Gamma (P.1) and Delta (B.1.617.2) – only harboured one to three mutations in the receptor-binding domain (RBD). However, in November 2021, the Omicron (B.1.1.529) variant emerged with 15 amino acid mutations in the RBD as compared to the wild-type strain. A series of studies showed that these mutations significantly increased viral transmissibility and immune evasion capabilities [1,4].

Subsequent to the dramatic changes that occurred in the RBD of Omicron, SARS-CoV-2 has continued to evolve into different lines of variants. The initial Omicron BA.1 (B.1.1.529) variant gave way to a series of subvariants that have dominated the pandemic at different times [4]. For example, the Omicron BA.5 subvariant was dominant during the latter half of 2022, while the XBB variant became the major subvariant in early 2023 [5]. In the latter half of 2023, XBB-related variants, such as EG.5.1, XBB.1.16 and HV.1, sequentially became the most prominent strains. Meanwhile, the new Omicron BA.2.86 variant attracted strong attention due to the large number of mutations in its spike protein. These mutations included 35 in the spike protein and 12 in the RBD, as compared with XBB.1.5 [6]. However, the prevalence of BA.2.86 did not surpass that of the XBB-related circulating variant, EG.5.1. Until early 2024, the Omicron BA.2.86 descendant variant, JN.1, which has only one additional substitution (L455S) in the RBD compared with BA.2.86, rapidly replaced HV.1 as the predominant variant [7,8]. Subsequently, JN.1-derived variants, KP.3.1.1, with additional substitutions in the RBD of JN.1 (F456L and Q493E) have shown enhanced immune evasion compared to the parental virus of the lineage [9,10]. Currently, the recombinant variant XEC, which is derived from KS.1.1 and KP.3, shows high potential to become the next dominant strain due to its rapid spread worldwide [10,11].

SARS-CoV-2 mRNA vaccines are robust and can be quickly altered to target current variants. However, not all individuals are eligible for vaccination. For instance, some immunocompromised individuals cannot be vaccinated due to cancer treatment, organ transplant, HIV status or congenital immunodeficiency. These individuals are still at high risk of severe disease, hospitalization and death due to SARS-CoV-2 [12,14]. Thus, there is a continued need for additional clinical treatments to protect unvaccinated individuals.

Monoclonal antibodies (mAbs) are highly specific and can be utilized as precise treatments for many diseases [15,16]. During the past four years, numerous mAbs have received US Emergency Use Authorization (EUA) for clinical treatment of SARS-CoV-2 [3]. These antibodies are designed to reduce disease severity by neutralizing SARS-CoV-2 via spike protein binding [17,18]. Importantly, SARS-CoV-2 infection is mediated by the spike protein, which is composed of an S1 and S2 domain. The S1 domain contains the N-terminal domain, RBD, subdomain 1 and subdomain 2 [19]. Viral entry occurs upon binding of the spike protein RBD to angiotensin-converting enzyme 2 (ACE2) at the host cell surface [20]. Thus, the RBD domain is a critical domain for virus infection, and it serves as a common target for vaccine design and neutralizing antibody development [21,22].

Among the various methodologies utilized for antibody generation, single B-cell sorting and cloning is widely considered to be a speedy and reliable approach [23,24]. Using this strategy, researchers can obtain a potential antibody candidate and identify lead sequences for further development within about 1 month [18]. As such, this approach has been used to accelerate antibody development for infectious diseases. Nearly all of the neutralizing antibodies receiving EUA approval during the coronavirus disease 2019 pandemic were developed using single B-cell cloning approach, including bamlanivimab, etesevimab, bebtelovimab, sotrovimab, cilgavimab and tixagevimab. In our previous study, we successfully developed potent and broadly neutralizing antibodies against different SARS-CoV-2 variants (Alpha to Omicron XBB.1.5) by performing human single B-cell sorting [25]. However, obstacles to this approach include the quick pace of virus evolution and limitations on the supply of human blood specimens. Thus, alternative efficient approaches for rapid antibody screening are still needed, especially those utilizing easily accessible animal models. In our previous studies, we immunized mice with mRNA encapsulated in lipid nanoparticles (mRNA-LNPs) and utilized traditional hybridoma fusion technology to generate a potent and broadly neutralizing antibody against SARS-CoV-2 strains ranging from Alpha to Omicron BA.1 [26,27]. In this study, we extended this work by combining mRNA-LNP immunization with single B-cell cloning to identify highly potent SARS-CoV-2-specific neutralizing antibodies. Using this approach, we isolated Omicron BA.5 and XBB.1.5 specific memory B cells from the splenocytes of immunized mice and then generated a set of chimeric antibodies (ChAbs), which were analysed for binding ability and neutralizing potency.

The most potent neutralizing antibodies specific to BA.5 and XBB.1.5 had respective IC_50_ values of 1.9 and 2.3 ng ml^−1^. We also identified XBB.1.5-ChAb-26 from XBB.1.5-immunized mouse splenocytes. This antibody could potently neutralize current pandemic variants, such as JN.1, KP.3.1.1 and XEC (respective IC_50_ values of 10.9, 21.4 and 23.6 ng ml^−1^). Binding of XBB.1.5-ChAb-26 to XBB.1.5 RBD protein was inhibited by mutations at sites Y453A and T500A. In contrast, the other two antibodies were inhibited by mutations at sites Y453 and R498. This difference may explain the retained neutralizing ability of XBB.1.5-ChAb-26 against JN.1-derived variants. Collectively, these data suggest that the combination of mRNA-LNP immunization with single B-cell antibody technology is a powerful approach for efficient identification of highly functional antibodies. Furthermore, this approach can provide an accessible method for antibody drug development against infectious diseases. These findings provide valuable information for the development of anti-SARS-CoV-2 antibody drugs that may contribute to clinical treatments and also yield fundamental knowledge about virus neutralization.

## Methods

### mRNA-LNP preparation

mRNA-LNPs encoding SARS-CoV-2 full-length spike (FLS) protein were prepared according to protocols outlined in our previous study [26]. Briefly, mRNA was synthesized by *in vitro* transcription, using a template that encodes the FLS protein of SARS-CoV-2 BA.5 or XBB.1.5 variants. The mRNA contained a 5′ UTR, IgG kappa leader sequence, a 3′ UTR and a poly(A) tail. Individual lipids were dissolved in ethanol and mixed containing ALC-0315 (MedChemExpress), 1, 2-distearoyl-sn-glycero-3-phosphocholine (Avanti Polar Lipids), cholesterol (Sigma-Aldrich) and ALC-0159 (MedChemExpress) with molar ratios of 46.3 : 9.4 : 42.7 : 1.6. The lipid mixture was combined with a 50-mM sodium acetate buffer pH 4.5, containing mRNA at a ratio of 3 : 1 (aqueous: ethanol), prior to mixing in NanoAssemblr^®^ IGNITE NxGen Cartridges (Precision NanoSystems Inc.). After encapsulation, mRNA-LNPs were dialysed against PBS (pH 7.4) and concentrated by Amicon Ultra-Centrifugal Filter with a 10 kDa cutoff (Merck). Reference sequences for SARS-CoV-2 variants were obtained from the NCBI Virus VSSI interface, filtered by PANGO lineage. Corresponding GenBank accession numbers for the spike proteins of all variants used in this study are listed in Table S1, available in the online Supplementary Material.

### Mouse immunization and splenocyte harvest

All animal experiments were approved by the Academia Sinica Institutional Animal Care and Use Committee at Academia Sinica, Taiwan (IACUC protocol no. 22-11-1921). Four to 6-week-old BALB/c female mice were immunized intramuscularly with 10 µg SARS-CoV-2 FLS mRNA-LNP at 2-week intervals for a total of four immunizations. Serum samples were collected after three immunizations, and splenocytes were harvested 7 days after the final boost. Red blood cells were lysed by incubation in eBioscience 1×RBC lysis buffer (Invitrogen) for 5 min at room temperature. The reaction was stopped with PBS, and the cells were centrifuged at 300 ***g*** for 5 min. After removing the supernatant, the splenocytes were resuspended in PBS containing 2% FBS for further single splenocyte isolation.

### RBD-specific B cell sorting

Cells were stained for 30 min on ice with APC-eFluor 780 (to identify live cells), APC rat anti-mouse CD19 (BD Biosciences), PE hamster anti-mouse CD3e (BD Biosciences), Brilliant Violet 510 goat anti-mouse IgG (Sony Biotechnology) and BV421(BD Biosciences)-conjugated RBD in PBS containing 2% FBS. After washing, the cells were resuspended in PBS with 2% FBS, and RBD-specific B cells (CD19^+^CD3^-^IgG^+^RBD^+^) were sorted into 96-well PCR plates containing 10 µl capture buffer (10 µl of 1 M Tris-HCl, pH 8.0, and 25 µl of RNasin in 1 ml RNase-free water) via BD FACSAria III (BD Biosciences). The plates were stored at −80 °C for further experiments.

### Construction and expression of antibodies

Single cells were thawed on ice and utilized for real-time PCR (QIAGEN OneStep RT-PCR Kit). Nested PCR was performed with the primers described previously [28,29]. After analysis of the VDJ sequence by IMGT, the V_H_ and V_K_ gene fragments were amplified with PCR and digested with appropriate restriction enzymes. The V_H_ genes were cloned into a modified expression vector with a signal peptide and the human constant region of IgG1. The V_K_ genes were cloned into a modified expression vector with a signal peptide and human kappa chain constant region. The V_H_ and V_K_ plasmids were transfected into Expi293F cells for antibody production. After 5 days in culture, individual antibodies were purified from the culture supernatant using protein G resin (GE Healthcare). The antibodies were exchanged to PBS buffer and analysed by SDS-PAGE.

### ELISA

ELISA plates were coated with 1 µg ml^−1^ RBD-His of SARS-CoV-2 WT or different variants in coating buffer (0.1 M NaHCO_3_, pH 8.6) at 4 °C overnight, followed by washing with PBS and blocking with PBS containing 1% BSA at room temperature for 2 h. After blocking, appropriate concentrations of mouse serum, antibody or expression medium were added to different wells for 2 h, and the plates were incubated at room temperature. Next, the wells were washed with PBST_0.1_ (PBS containing 0.1% Tween-20) three times. Horseradish peroxidase-conjugated anti-human IgG (1 : 5,000) or anti-mouse IgG (H and L) was added, and plates were incubated for 1 h at room temperature. After three washes with PBST_0.1_, signal was produced using TMB solution (TM1999, Scytek Laboratories). The reaction was stopped with TMB Stop Buffer (TSB999, Scytek Laboratories), and absorbance was measured at 450 nm using an ELISA reader (VersaMax Tunable Microplate Reader; Molecular Devices).

### Pseudovirus neutralization assay

Pseudovirus neutralization assays were performed using HEK293T cells that stably expressed human ACE2 (HEK293T/hACE2). The different variants of SARS-CoV-2 pseudotyped lentivirus expressing full-length spike protein were provided by the National RNAi Core Facility (Academia Sinica, Taiwan). The HEK293T/hACE2 cells were seeded in 96-well white plates (Corning Costar) at a density of 1×10^4^ cells per well and cultured at 37 °C for 24 h. Various concentrations of antibodies (twofold dilution of ChAb-containing expression medium for primary screening, Fig. 2b) were pre-incubated with different SARS-CoV-2 variant pseudovirus strains at 1000 TU/well in 96-well plates. The mixtures were incubated at 37 °C for 1 h and then added to pre-seeded HEK293T/hACE2 cells. After a 24 h incubation at 37 °C, supernatants were replaced with DMEM+10% FBS for an additional 48 h. Next, ONE-Glo™ luciferase reagent (Promega) was added to each well for 3 min. Luminescence was measured with a microplate spectrophotometer (Molecular Devices). Negative (0% inhibition) and positive (100% inhibition) controls were, respectively, calculated using wells with pseudovirus only and cells only. The half-maximal inhibitory concentration (IC_50_) was calculated by performing nonlinear regression using Prism software version 9 (GraphPad Software Inc.). The average IC_50_ value for each antibody was determined from at least two independent experiments.

**Fig. 1.**
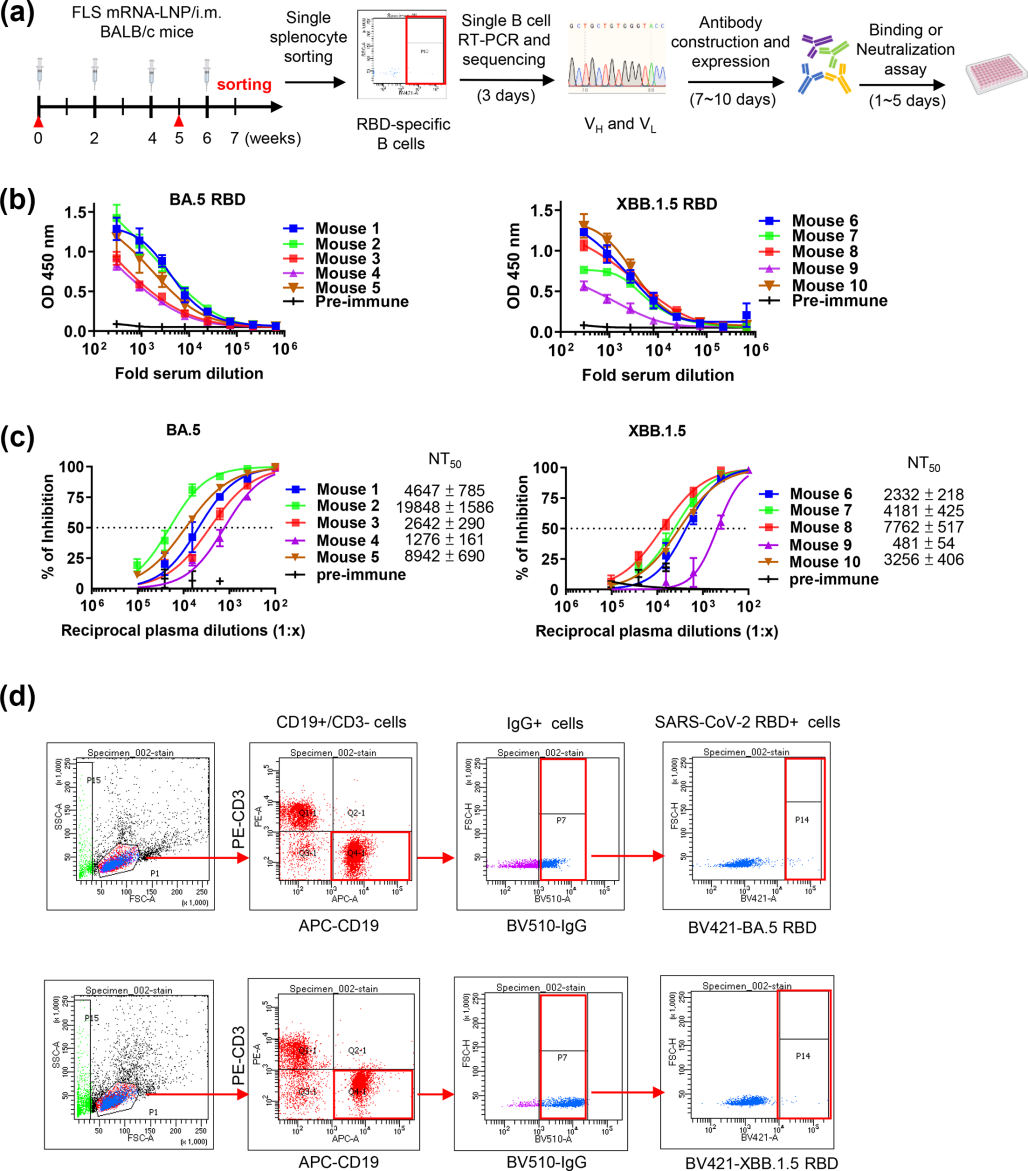
Isolation of single B cells targeting SARS-CoV-2 BA.5 or XBB.1.5-RBD from immunized mice. (a) Overview of experimental design and immunization schedule. BALB/c mice were immunized intramuscularly with FLS mRNA-LNPs at the indicated time points. The red triangle indicates blood draws. CD19^+^CD3^-^IgG^+^RBD^+^ B cells from mice immunized against FLS using mRNA-LNPs were sorted by flow cytometry. V_H_ and V_L_ fragments in single B cells were amplified and sequenced. The resulting constructs were transfected into Expi293F cells for antibody production, followed by binding and neutralization assays. (b) Binding activities of immunized mouse sera as determined by ELISA. The sera were incubated in ELISA plates coated with 1 µg ml^−1^ recombinant BA.5 or XBB.1.5-RBD protein, followed by detection with HRP-conjugated anti-mouse antibody. (c) Neutralization assay of SARS-CoV-2 BA.5 or XBB.1.5 pseudovirus with immunized mouse serum. (d) The gating strategy for BA.5 or XBB.1.5-RBD-specific B cells from immunized mice. From left to right, the panels show gating strategies used to isolate lymphocytes, APC-CD19^+^PE-CD3^-^ cells (Q4-1 population) and CD19^+^CD3^-^ cells, which were further gated for BV510-IgG and BV421-BA.5 or XBB.1.5-RBD.

### Cellular ELISA

HEK293T cells were transiently transfected with BA.5 RBD-His, XBB.1.5 RBD-His or related mutant RBD-His plasmids in 6-well plates. The next day, the cells were seeded in 96-well plates. The cells were fixed in 4% paraformaldehyde in PBS for 45 min at room temperature, 48 h after transfection. Fixed cells were permeabilized in 0.1% Triton X-100 at room temperature for 30 min. After blocking with 5% milk, antibodies against RBD were added to the wells (1 µg ml^−1^) for 1 h at room temperature. After washing, HRP-conjugated anti-human antibody (Jackson ImmunoResearch) (1 : 2,000 dilution) or HRP-conjugated anti-His-tag antibody (GeneTex) (1 : 2,000 dilution) was added for 1 h at room temperature. Excess secondary antibody was washed off, and then the signal was produced using TMB solution (TM1999). The reaction was stopped with TMB Stop Buffer (TSB999), and absorbance was measured at 450 nm by an ELISA reader (VersaMax Tunable Microplate Reader). The binding signal was normalized to the corresponding anti-His-tag antibody signal in order to account for differences in protein expression levels.

## Results

### Isolation of SARS-CoV-2-BA.5 and XBB.1.5-specific B cells from immunized mice

To generate specific antibodies that bind to SARS-CoV-2 BA.5 and XBB.1.5 RBD protein, we immunized BALB/c mice with LNP-encapsulated mRNA encoding either BA.5 or XBB.1.5 spike protein and then performed RT-PCR on single B cells, as outlined in Fig. 1a. The mice were immunized with mRNA-LNPs using a protocol developed for our previous study [26]. Serum samples were collected following three inoculations, and splenocytes were harvested after the final boost. The serum samples were analysed for RBD binding levels by ELISA. Serum samples from all immunized mice showed binding to SARS-CoV-2 BA.5 RBD or SARS-CoV-2 XBB.1.5 RBD (Fig. 1b). The neutralizing capacity of the serum was then analysed with a SARS-CoV-2 BA.5 or XBB.1.5 pseudovirus assay. All serum samples exhibited potent neutralizing ability. The range of NT_50_ values was from 1/1,276 to 1/19,848 for sera from SARS-CoV-2 BA.5 immunized mice. The range was from 1/481 to 1/7,762 for sera from SARS-CoV-2 XBB.1.5 immunized mice (Fig. 1c). The mice from which serum samples with the most potent neutralizing ability were isolated were further utilized for isolation of neutralizing antibodies. Using Fluorescence Activated Cell Sorting, CD19-positive, CD3-negative, IgG-positive and SARS-CoV-2 BA.5- or XBB.1.5 RBD-bound-specific B cells were individually isolated (Fig. 1d). The V_H_ and V_L_ regions of selected single B cells were amplified with specific primers [28,29], and the VDJ sequences were analysed by IMGT. Using this approach, we successfully constructed 42 chimeric antibody clones from SARS-CoV-2 BA.5 RBD-targeting B cells and 24 chimeric antibody clones from SARS-CoV-2 XBB.1.5 RBD-targeting B cells.

**Fig. 2.**
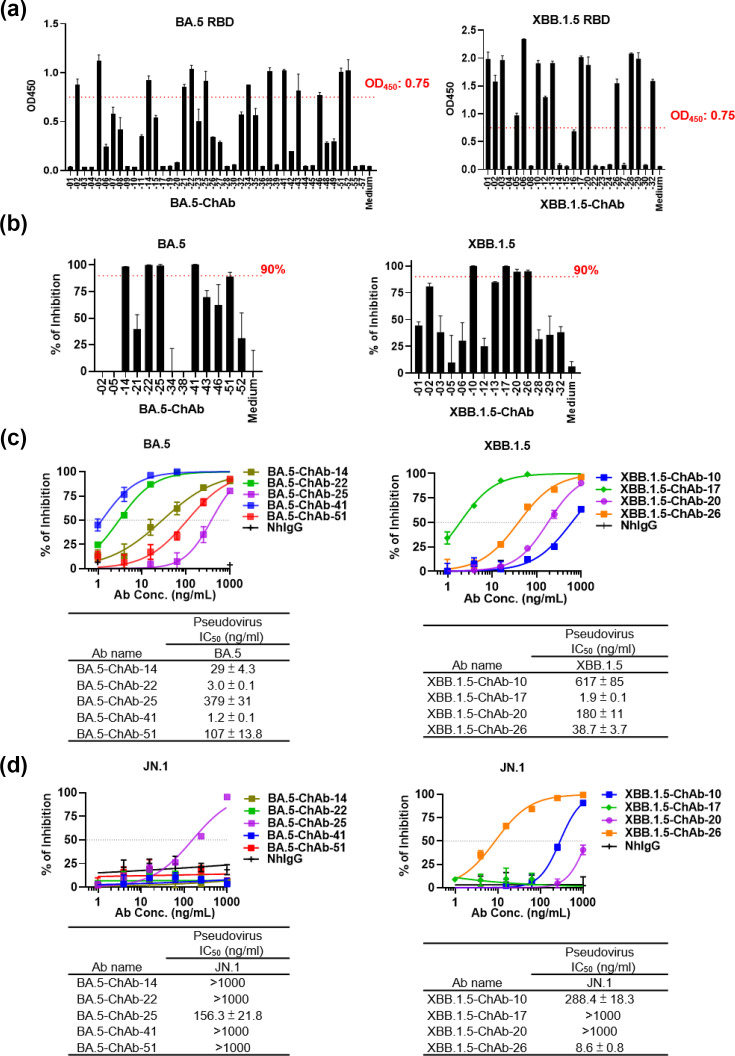
Identification of SARS-CoV-2 Omicron BA.5- and XBB.1.5-neutralizing antibodies derived from single B cells of immunized mice. (a) ELISA was performed to screen the BA.5 and XBB.1.5 RBDbinding antibodies. Plates were coated with BA.5 or XBB.1.5 RBD protein (1 µg ml^−1^) and incubated with a twofold dilution of ChAb expression medium. Medium of non-transfected cells was used as a negative control. (b) A neutralization assay was performed to screen for BA.5 and XBB.1.5- antibodies using a twofold dilution of ChAb-containing expression medium. Neutralization activity is expressed as a percentage of inhibition relative to virus-only controls. (c) Five BA.5 and four XBB.1.5 antibodies were purified and examined. The IC_50_ (concentration of half inhibition) was measured using a pseudotype virus neutralization assay. (d) Neutralization activities of BA.5-ChAbs and XBB.1.5-ChAbs against pseudoviruses of JN.1.

### Screening of neutralizing antibodies against SARS-CoV-2 BA.5 and XBB.1.5 from Expi293F cell expression media

We screened the binding ability of 42 anti-SARS-CoV-2 BA.5 ChAbs and 24 anti-SARS-CoV-2 XBB.1.5 ChAbs by performing ELISA on media of Expi293F cells that express each ChAb (Fig. 2a). The ChAb expression media that showed potent binding ability (OD_450_ above 0.75) were further assessed with neutralizing assays (Fig. 2b). Based on the results, five BA.5-ChAbs and four XBB.1.5-ChAbs with neutralizing ability above 90% were chosen for further study. We purified the ChAbs of these clones and evaluated their neutralizing abilities against Omicron BA.5 and XBB.1.5 pseudotype viruses. Pseudovirus neutralizing assays showed that the neutralizing potencies of the five BA.5-ChAbs (against BA.5) and four XBB.1.5-ChAbs (against XBB.1.5) had IC_50_ values, respectively, ranging from 1.2 to 379 ng ml^−1^ and from 1.9 to 617 ng ml^−1^. Among the tested antibodies, BA.5-ChAb-41 and XBB.1.5-ChAb-17 exhibited the most potent neutralizing abilities against BA.5 and XBB.1.5, with IC_50_ values of 1.2 and 1.9 ng ml^−1^, respectively (Fig. 2c). With the continual evolution of SARS-CoV-2, the JN.1 subvariant became a major variant in the first half of 2024. To determine whether antibodies generated against earlier Omicron lineages retained activity against this new dominant variant, we retrospectively assessed the neutralizing capabilities of the existing five BA.5-ChAbs and four XBB.1.5 ChAbs against the JN.1 pseudotype virus. In this secondary examination, BA.5-ChAb-25, XBB.1.5-ChAb-10 and XBB.1.5-ChAb-26 exhibited neutralizing ability against JN.1, with XBB.1.5-ChAb-26 exhibiting the most potent neutralizing ability (Fig. 2d).

### Neutralizing abilities of BA.5-ChAb-41, XBB.1.5-ChAb-17 and XBB.1.5-ChAb-26 against previous and current variants

**Fig. 3.**
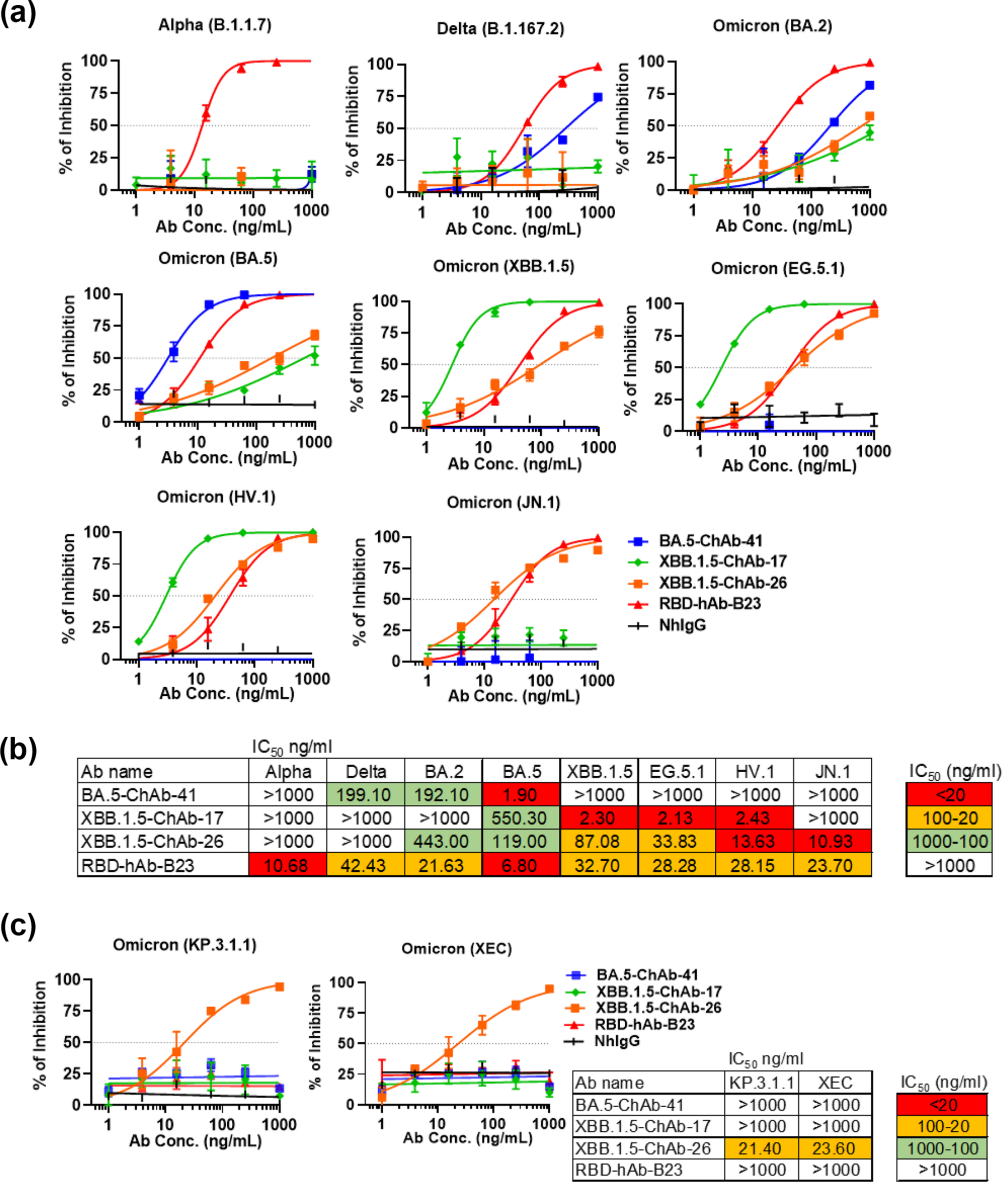
Neutralizing activities of BA.5-ChAbs and XBB.1.5-ChAbs towards SARS-CoV-2 pseudotyped variants. (**a**) Neutralization activities of BA.5-ChAb-41, XBB.1.5-ChAb-17, XBB.1.5-ChAb-26 and previously published RBD-hAb-B23 were measured against pseudoviruses of different SARS-CoV-2 subvariants (Alpha, Delta, Omicron BA.2, BA.5, XBB.1.5, EG.5.1, HV.1 and JN.1). (**b**) The heatmap shows the pseudovirus neutralization activities for the antibodies in (a). Each value is the average of three independent experiments. The colours indicate IC_50_: red, < 20 ng ml^−1^; yellow, 20–100 ng ml^−1^; green 100–1,000 ng ml^−1^. (**c**) Neutralization activities of BA.5-ChAb-41, XBB.1.5-ChAb-17, XBB.1.5-ChAb-26 and RBD-hAb-B23 against pseudoviruses of current SARS-CoV-2 subvariants, KP.3.1.1 and XEC. Each value is the average of two independent experiments.

Based on our primary screening experiments, BA.5-ChAb-41, XBB.1.5-ChAb-17 and XBB.1.5-ChAb-26 were identified as the most potent neutralizing antibodies against BA.5, XBB.1.5 and JN.1. Because each antibody represented the best performing candidate against a distinct predominant variant at different stages of the pandemic, we next tested the breadth of neutralization for each of the three antibodies. To this end, we re-evaluated the neutralization profiles of the three identified antibodies, along with the human antibody RBD-hAb-B23 from our previous study [25], across a panel of SARS-CoV-2 subvariants. The panel of subvariants includes Alpha, Delta, Omicron BA.2, BA.5, XBB.1.5, EG.5.1, HV.1 and JN.1 (Fig. 3a, b). BA.5-ChAb-41 showed the lowest IC_50_ against BA.5, and it had moderate IC_50_ values when tested against Delta and BA.2 variants. However, it lacked neutralizing activity against Alpha, XBB.1.5, EG.5.1, HV.1 and JN.1. XBB.1.5-ChAb-17 exhibited potent neutralizing abilities against XBB.1.5, EG.5.1 and HV.1 (IC_50_ values ranging from 2.13 to 2.43 ng ml^−1^). In addition, XBB.1.5-ChAb-26 not only showed moderate neutralizing ability towards XBB.1.5 (IC_50_ of 87.08 ng ml^−1^), but it also displayed the most potent neutralizing activity against the current variant, JN.1 (IC_50_ of 10.93 ng ml^−1^ from three independent experiments). Furthermore, RBD-hAb-B23 exhibited neutralizing ability towards current pandemic variants, EG.5.1, HV.1 and JN.1 (Fig. 3a, b). In light of the ongoing emergence of new subvariants, we next assessed the neutralizing capabilities of BA.5-ChAb-41, XBB.1.5-ChAb-17, XBB.1.5-ChAb-26 and RBD-hAb-B23 against KP.3.1.1 and XEC pseudotyped viruses. Among the tested antibodies, only XBB.1.5-ChAb-26 exhibited potent naturalizing activity, with low IC_50_ values of 21.4 ng ml^−1^ for KP.3.1.1 and 23.6 ng ml^−1^ for XEC (Fig. 3c).

### Binding activities and epitopes of BA.5-ChAb-41, XBB.1.5-ChAb-17 and XBB.1.5-ChAb-26 across selected SARS-CoV-2 variants

**Fig. 4.**
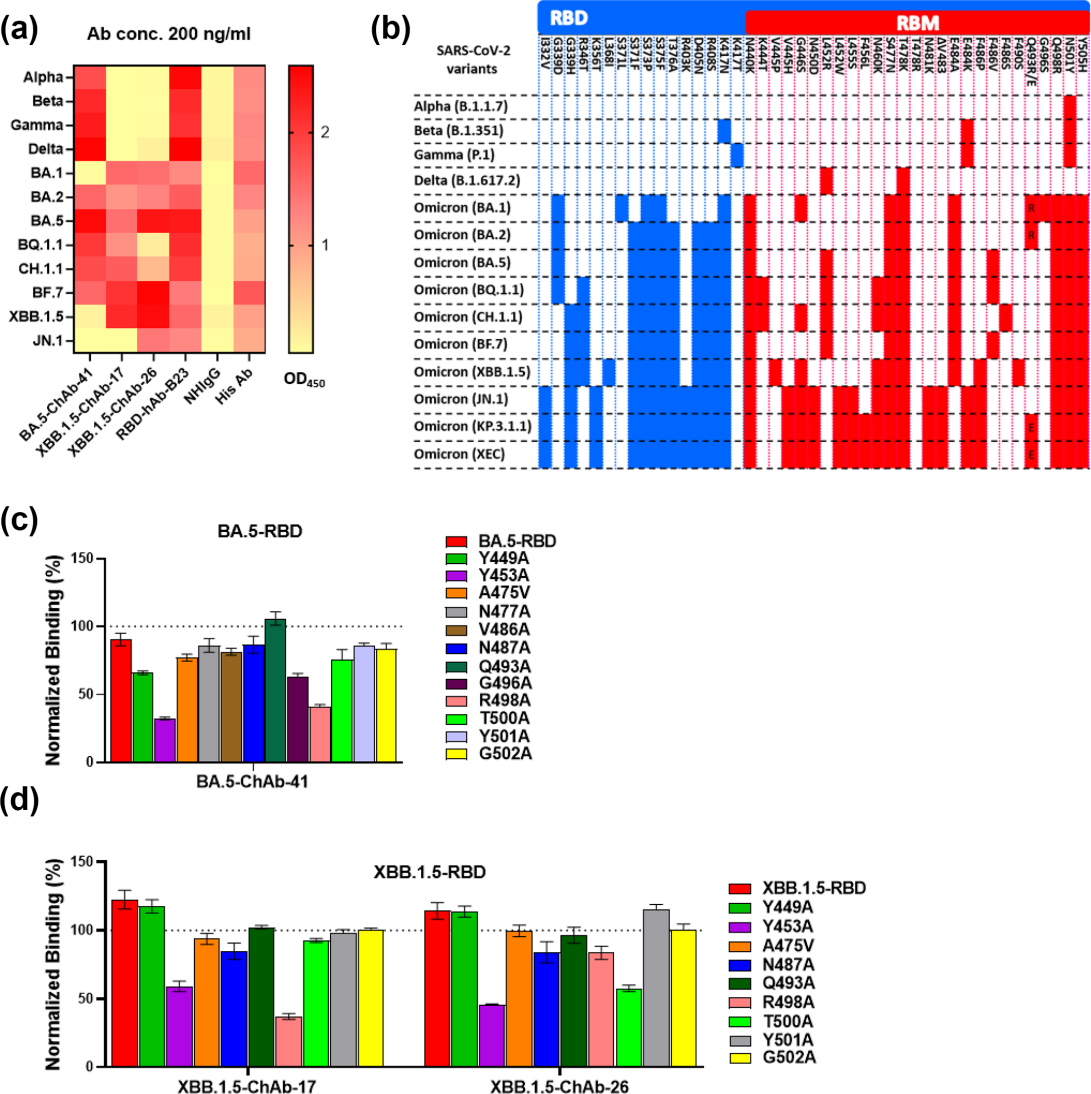
Examination of RBD-binding ability and epitope mapping of BA.5-ChAb-41, XBB.1.5-ChAb-17 and XBB.1.5-ChAb-26. (**a**) Binding activity of four RBD-ChAbs and one broadly neutralizing human antibody against RBDs of Alpha, Beta, Gamma, Delta, Omicron BA.1, BA.2, BA.5, BQ.1.1, Ch.1.1, BF.7, XBB.1.5 and JN.1, according to ELISA. Anti-6×His Ab was used as a positive control. Normal human IgG (NhIgG) was used as a negative control. (**b**) Summary of amino acid substitutions in RBD regions of Alpha, Beta, Gamma, Delta, BA.1, BA.2, BA.5, BQ.1.1, CH.1.1, BF.7, XBB.1.5, JN.1, KP.3.1.1 and XEC relative to the original SARS-CoV-2 wild-type strain. (**c**) and (**d**) Epitope mapping by site-directed mutagenesis assay. HEK293T cells transiently expressed exogenous BA.5-RBD-His, XBB.1.5-RBD-His or mutant RBD-His proteins with single alanine or valine mutations. The binding of RBD-ChAbs to the RBD mutants was examined by cellular ELISA. The results are normalized to the corresponding anti-His-tag antibody signal and compared with the parental RBD.

To investigate whether the three RBD-ChAbs share overlapping epitopes, we carried out ELISA with RBD proteins containing mutations corresponding to selected variants (Fig. 4a). When tested against Alpha, Beta, Gamma, Delta, Omicron BA.1, BA.2, BA.5, BQ.1.1, CH.1.1, BF.7, XBB.1.5 and JN.1, the three ChAbs showed distinct binding patterns. BA.5-ChAb-41 exhibited strong binding with earlier VOCs and major Omicron subvariants, but it lost binding ability against BA.1, XBB.1.5 and JN.1. XBB.1.5-ChAb-17 and XBB.1.5-ChAb-26 could not bind any RBD of earlier VOCs, but the antibodies bound to most Omicron sublineages. XBB.1.5-ChAb-17 lost binding against JN.1, while XBB.1.5-ChAb-26 retained binding and neutralizing ability to JN.1 (Figs3b  4a).

While Omicron and WT RBDs exhibit similar ACE2 receptor-binding characteristics, a novel interaction has been reported to occur at distinct sites on the Omicron RBD [30,32]. To further investigate the critical residues involved in ChAb binding, we modified candidate positions within the receptor-binding motif (RBM) based on previous structural and functional studies [30,32]. Accordingly, we individually mutated 12 residues of BA.5-RBD (Y449A, Y453A, A475V, N477A, V486A, N487A, Q493A, G496A, R498A, T500A, Y501A and G502A). Similarly, we individually changed nine residues (Y449A, Y453A, A475V, N487A, Q493A, R498A, T500A, Y501A and G502A) of XBB.1.5-RBD to alanine or valine. The mutant RBDs were transiently expressed in HEK293T cells, and cellular ELISAs were performed to assess the impact of each mutated residue on the binding of the three ChAbs (Fig. 4c, d). The results revealed that a single mutation at either Y453A or R498A dramatically decreased the binding of BA.5-ChAb-41 and XBB.1.5-ChAb-17 by ~50–60%. Single Y453A or T500A mutations in XBB.1.5 RBD attenuated the binding of XBB.1.5-ChAb-26 (by about 50%). These data suggest that the epitopes recognized by the three ChAbs in RBD may differ, reflecting the differential neutralizing ability of XBB.1.5-ChAb26 towards JN.1-related variants.

## Discussion

Although current anti-SARS-CoV-2 mRNA vaccines are robust and well-developed, there remains a significant clinical need for additional therapeutic options for SARS-CoV-2-infected individuals. This need is particularly urgent for high-risk populations such as immunocompromised individuals. mAbs are widely used to treat various diseases [15] and may be highly beneficial for clinical treatment of SARS-CoV-2 infection in this high-risk population. Here, we combined a single B-cell cloning approach with mRNA-LNP immunization and successfully identified specific SARS-CoV-2 neutralizing antibodies within a short time-frame of just 10 weeks from mouse immunization to antibody identification. We used the culture supernatant from Expi293F cells transfected with the identified antibody clones to quickly select those with the highest binding and neutralizing potency. By this approach, we identified five neutralizing antibodies against the BA.5 variant and four against the XBB.1.5 variant, with IC_50_ values ranging from 1.2 to 617 ng ml^−1^ (Fig. 2c). These findings underscore the efficiency of this platform for screening functional antibodies. Importantly, the utilization of a mouse model in this approach offers an attractive strategy for antibody drug development that may be applied for future pandemic infectious diseases. The approach may be particularly beneficial during the early stages of a pandemic, when human samples are difficult to obtain. The generation of highly potent broadly neutralizing antibodies is often associated with prolonged germinal centre reactions and extensive somatic hypermutation (SHM). In this study, sequence analysis of the isolated antibodies revealed low to moderate levels of SHM, with VH germline identities ranging from 81.4 to 95.9% (Table S2), similar to our previously screened antibodies [26,33]. Two studies in mice have shown that germinal centre responses and SHM are initiated rapidly following mRNA vaccination, typically within 1–2 weeks after immunization [34,35], suggesting that early affinity maturation occurs within a relatively short time frame. However, the full extent of SHM and affinity maturation achievable under our immunization schedule cannot be definitively determined in this study.

Over the past 5 years, numerous mAbs have received EUAs for clinical treatment of SARS-CoV-2. However, the Omicron variant emerged with extensive mutations in the RBD, which caused nearly all of the approved mAbs to lose therapeutic potency and led to their withdrawal from clinical use [36,38]. Sotrovimab retains weak neutralizing ability against recent subvariants, such as XBB.1.5, XBB.1.16.1 and EG.5.1.3, but it has lost efficacy against BA.2.86.1, JN.1 and KP.3 [39,41]. Recently, several antibodies under development [i.e. SA55 (Phase 2, NCT06042764), VYD222 (Phase 3, NCT06039449), AZD3152 (Phase 2/3, NCT05648110), BD55-1205, CYFN1006-1 and CYFN1006-2] have demonstrated broad neutralization against current variants [42,45]. In particular, SA55, CYFN1006-1 and CYFN1006-2 demonstrated broadly potent neutralizing activity across variants from ancestral strain to XEC, with reported IC_50_ values below 10 ng ml^−1^ [43]. BD55-1205 exhibited strong neutralization against recent subvariants, including JN.1 and KP.3 [45]. VYD222 showed potent neutralizing activity against WT and Delta (IC_50_ : 8.4 and 5.2 ng ml^−1^), but moderate against JN.1 and KP.3 [44] (IC_50_ : 74.6 ng ml^−1^ and 223 ng ml^−1^). Thus, these antibodies represent promising therapeutic candidates for currently circulating SARS-CoV-2 variants. In this study, we showed that XBB.1.5-ChAb-26 has robust neutralizing ability against a broad spectrum of circulating subvariants, including XBB.1.5, EG.5.1, HV.1, JN.1, KP.3.1.1 and XEC (Fig. 3). Although both BA.5-ChAb-25 and XBB.1.5-ChAb-10 exhibited moderate activity against JN.1, BA.5-ChAb-25 lost neutralization against other subvariants (XBB.1.5, KP.3.1.1 and XEC). Meanwhile, XBB.1.5-ChAb-10 exhibited limited activity against XBB.1.5 and BA.5 (Fig. 2c and S1). Finally, XBB.1.5-ChAb-26 exhibited a potent neutralization against recent Omicron subvariants, but not previous strains.

The mechanism of action for SARS-CoV-2 neutralizing antibodies involves protecting cells from viral infection through blocking the interaction between the spike protein and the ACE2 receptor. However, the Omicron BA.1 variant has 32 mutations in the spike protein, including 15 mutations in the RBD (G339D, S371L, S373P, S375F, K417N, N440K, G446S, S477N, T478K, E484A, Q493K/R, G496S, Q498R, N501Y and Y505H) and 10 in the RBM for ACE2 binding (Fig. 4b). These mutations change the structure and charge of the spike protein surface, affecting the sites at which neutralizing antibodies would typically bind. Thus, vaccines and antibodies developed to target previous variants have lost their protective activities. For instance, the K417N and Q493 mutations have rendered etesevimab ineffective, while E484A and Q493R mutations diminished bamlanivimab’s neutralizing potency [46,48]. Similarly, N440K and G446S mutations contribute to immune escape from imdevimab [49]. Furthermore, the multiple mutations S477N/T478K/E484A on Omicron BA.1 significantly reduce the effectiveness of tixagevimab [50]. All of these neutralizing antibodies were rendered ineffective due to mutations, which resulted in the withdrawal of their respective EUAs.

Here, we showed that the Y453 and T500 are likely epitopes of XBB.1.5-ChAb-26, which exhibits neutralizing ability against current pandemic variants, JN.1, KP.3.1.1 and XEC. A recent structural analysis indicated that ten residues on BA.2.86 RBD interact with ACE2, specifically the side chains of N417, Y449, Y453, Y489, Q493, R498, T500, T501, and the main chain of A475 and G502. The Y449, N477, N487, S494, R498, T500 and G502 in JN.1 [51]. Because the RBD interacts with ACE2, it is possible that XBB.1.5-ChAb-26 may exert its neutralizing effect on JN.1 by binding to T500, thereby disrupting the interaction between the JN.1 RBD and ACE2. In addition, the heavy chain of CYFN1006-1 forms hydrogen bonds with the conserved residue of T500 in the RBD, locking the RBD in the ‘down’ conformation and potentially contributing to its broadly neutralizing activity [43]. However, interactions involving a single residue are unlikely to fully explain the observed neutralization breadth, as overall potency is more likely determined by the contributions of the entire binding interface. The precise binding between XBB.1.5-ChAb-26 and JN.1 RBD will need to be determined by methods such as X-ray crystallography or cryo-electron microscopy (cryo-EM).

In conclusion, we employed mRNA-LNP immunization with single B-cell antibody technology in a mouse model to rapidly identify anti-BA.5 and anti-XBB.1.5 neutralizing antibodies. Among the identified antibodies, XBB.1.5-ChAb-26 exhibited remarkable neutralizing activity against pseudotyped viruses of currently circulating strains, including JN.1, KP.3.1.1 and XEC. We anticipate that the strategy we used for this study will not only accelerate antibody drug development, but will also provide a valuable platform for basic research on infectious diseases.

## Supplementary material

10.1099/jgv.0.002251 Uncited Supplementary Material 1.

## Abbreviations

ACE2: angiotensin-converting enzyme 2
ChAb: chimeric antibody
Cryo-EM: Cryo-electron microscopy
EUA: Emergency Use Authorization
FLS: full-length spike
IC_50_: half maximal inhibitory concentration
LNP: lipid nanoparticle
mAb: monoclonal antibody
NT50: half-maximal neutralizing antibody titre
RBD: receptor-binding domain
RBM: receptor-binding motif
SARS-CoV-2: severe acute respiratory syndrome coronavirus 2
SHM: somatic hypermutation
VOC: variant of concern
