# Supplementary material for: Broadly neutralizing monoclonal antibodies derived from mRNA LNP immunization exhibit potent neutralizing ability against JN.1, KP.3.1.1 and XEC new Omicron variants
Source: J Gen Virol. 2026 Apr 24;107(4):002251. doi: 10.1099/jgv.0.002251 (PMC13131021; doi:10.1099/jgv.0.002251)
Supplement: Uncited Supplementary Material 1. [file jgv-107-02251-s001.pdf]

Figure S1

A

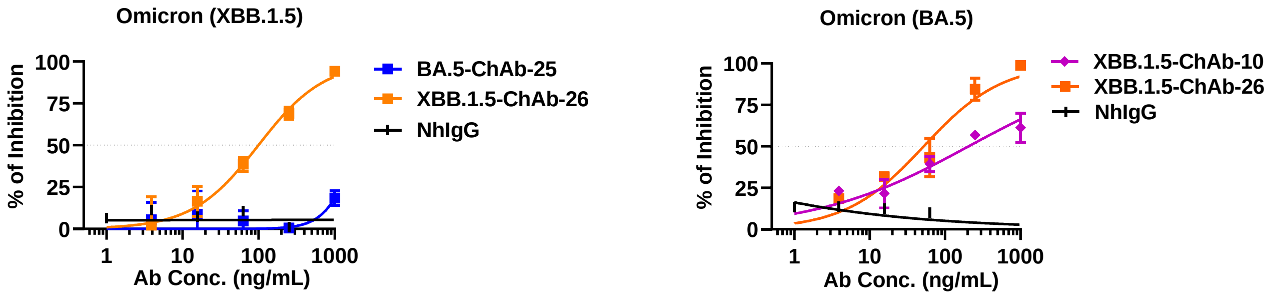

	Pseudovirus IC_{50} (ng/ml)
Ab name	XBB.1.5
BA.5-ChAb-25	>1000
XBB.1.5-ChAb-26	99.4 ± 13.6

	Pseudovirus IC_{50} (ng/ml)
Ab name	BA.5
XBB.1.5-ChAb-10	204.3 ± 50
XBB.1.5-ChAb-26	52.0 ± 9.9

B

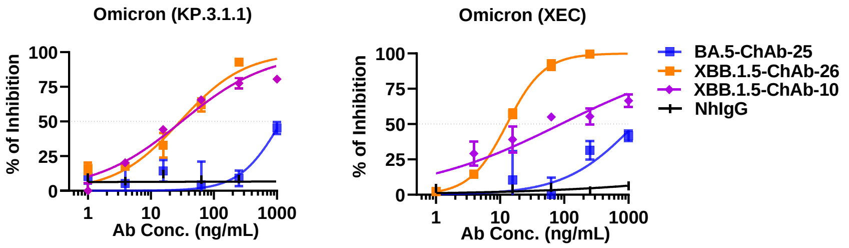

	Pseudovirus IC_{50} (ng/ml)	
Ab name	KP.3.1.1	XEC
BA.5-ChAb-25	>1000	>1000
XBB.1.5-ChAb-10	37.5 ± 7.3	88.4 ± 30
XBB.1.5-ChAb-26	29.1 ± 4.0	12.8 ± 0.4

Figure S1. Neutralizing activities of BA.5-ChAb-25 and XBB.1.5-ChAb-10 against SARS-CoV-2 pseudotyped variants, XBB.1.5, BA.5, KP.3.1.1, and XEC. (A) Cross-neutralization activities of BA.5-ChAb-25 against XBB.1.5, and XBB.1.5-ChAb-10 against BA.5 pseudoviruses. (B) Cross-neutralization activities of BA.5-ChAb-25, XBB.1.5-ChAb-10, and XBB.1.5-ChAb-26 neutralizing antibodies against KP.3.1.1 and XEC.

Supplementary Table1. GenBank accession numbers of Surface glycoprotein (Spike protein) of SARS-CoV-2 variants used in this study. The table lists the reference sequences for spike proteins corresponding to each variant, including those used for mRNA vaccine construction, and pseudotyped neutralization assays.

Strain	GenBank accession number
B.1.1.7	QQH18545.1
B.1.617.2	QUQ00281.1
BA.2	UIR81940.1
BA.5	UQE51418.1
XBB.1.5	UZG29433.1
EG.5.1	WGP26425.1
HV.1	WKK53590.1
JN.1	WOH32760.1
KP.3.1.1	XCD02655.1
XEC	XEL39601.1

Supplementary Table2. Germline sequence identity of BA.5- and XBB.1.5-ChAbs. Heavy (H) and light (L) chain variable region genes were analyzed using IMGT/V-QUEST database.

Ab name	Chain	V gene	J gene	Germline identity (%)
BA.5-ChAb-14	H	IGHV1-9*02	IGHJ3*01	94.9
BA.5-ChAb-14	L	IGKV3-2*01	IGKJ1*01	98.0
BA.5-ChAb-22	H	IGHV1-9*02	IGHJ2*01	92.9
BA.5-ChAb-22	L	IGKV3-10*01	IGKJ1*02	90.9
BA.5-ChAb-25	H	IGHV1-47-48*01	IGHJ2*01	95.9
BA.5-ChAb-25	L	IGKV12-46*01	IGKJ2*01	94.7
BA.5-ChAb-41	H	IGHV1-47-28*01	IGHJ3*01	89.6
BA.5-ChAb-41	L	IGKV3-10*01	IGKJ5*01	92.9
BA.5-ChAb-51	H	IGHV1-47-14*01	IGHJ3*01	92.8
BA.5-ChAb-51	L	IGKV3-10*01	IGKJ1*01	98.0
XBB.1.5-ChAb-10	H	IGHV1-47-14*01	IGHJ4*01	93.9
XBB.1.5-ChAb-10	L	IGKV4-55*01	IGKJ4*02	91.6
XBB.1.5-ChAb-17	H	IGHV1-15*02	IGHJ3*01	81.4
XBB.1.5-ChAb-17	L	IGKV14-111*01	IGKJ5*01	90.5
XBB.1.5-ChAb-20	H	IGHV1-14*02	IGHJ3*01	94.9
XBB.1.5-ChAb-20	L	IGKV3-10*01	IGKJ4*01	93.9
XBB.1.5-ChAb-26	H	IGHV1-18-7*01	IGHJ2*01	86.7
XBB.1.5-ChAb-26	L	IGKV3-5*01	IGKJ1*01	91.9
